# Results of the Examination of the Stools of 422 Cases Admitted to a Cairo Hospital (March to August, 1916)

**Published:** 1918-05

**Authors:** 


***Results of the Examination of the Stools of 422 Cases Admitted to a Cairo Hospital (March to August, 1916).*** By Lieut.-Col. C. J. MARTIN, A. A. M. C., Capt. KELLAWAY, A. A. M. C., and Sister F. E. WILLIAMS, A. N. S. Abs. from the *Jour. R. A. M. C.*, Vol. XXX, N° 1, p. 101, Jan. 1918.

Most of the cases were beyond the acute stage of the disease and passed merely loose motions; but 217 contained mucopus with or without blood. No ground was discovered for supposing that any protozoological parasite other than Amoeba hystolytica was responsible for ill health.

Two organisms isolated culturally and biochemically resembled the Flexner group of bacilli, but failed to agglutinate with any Y or Flexner serum. Six months later 5 out of 6 strains tested agglutinated well.

